# Inhalation in Whooping Cough

**Published:** 1889-09

**Authors:** Elias J. Beall

**Affiliations:** Fort Worth, Tex.


### CORRESPONDENCE.

### INHALATION IN WHOOPING COUGH.

FORT WORTH, Tex., Aug., 19, 1889.

*Ed. Daniel's Texas Medical Journal:*

Some years ago I called the attention of the profession to the treatment of whooping-cough by inhalation. The mode suggested was easy in its technique, and well adapted to the comprehension of almost any one. The plan consisted of securing (with safety-pins) upon the breast, just below the chin and *outside* of the child's clothing, a pad of flannel, three or four ply, size, three and one-half by four inches. As a protection to the clothing of the child, the flannel pad should have a piece of oil cloth interposed between it and the clothing. That the sufferer be subjected to the constant influence of inspired air continuously medicated, the pad was occasionally moistened with the following mixture:

℞ Olei Sassafras,

Olei Terebinthinaæ, aa ʒiij.

Ext. Belladonnæ, Fluidi, ʒss.

Phenol-sodique, ad℥jj.

M.

With the above treatment I had good results in securing well marked amelioration of the paroxysms, if not shortening the duration of the disease.

During an epidemic of that malady through which the unprotected children of my *clientelle* passed, in the early part of this year, I had a corroboration of the good results of past years; and as I changed the medication somewhat, I take the liberty to again direct the attention of the profession to the treatment, and the modification further experience has induced me to make in the therapy employed. I now use:

| | | |
|---|---|---|
| ℞ | Thymol, | ℈j. |
| | Acidi carbolici, | ʒss. |
| | Olei sassafras, | |
| | Olei eucalypti, | aa fʒij. |
| | Olei piscis liquidæ, | |
| | Olei terebinthinæ, | |
| | Etheris, | fʒiv. |
| | Alcoholis, | tof℥iij. |
| M. | | |

Sig. Moisten pad every two or three hours with one-half teaspoonful, more or less. Keep vial well stopped.

In very aggravated cases of the disease I have sometimes supplemented the inhalation treatment by the occasional administration of the following combination:

| | | |
|---|---|---|
| ℞ | Acidi carbolici, | grs. iij. |
| | Sodii bromidi, | ℈ijss. |
| | Tr. belladonæ, | gtts. xx. |
| | Glycerini, | tʒiij. |
| | Aquæ, | tof℥ij. |
| M. | | |

Sig. Teaspoonful for child three or four years of age.

So frequently have I received expressions of satisfaction with this treatment; so frequently have I known the bottle "passed around among the neighbors;" and as the treatment is predicated upon the hypothesis maintained by some as to the nature of the disease, I again ask you to enclose the article in your *red bound jewel*, and send it out for the good I think it may carry to the little ones, physically, and to the doctors, mentally. Yours truly,

ELIAS J. BEALL, M. D.

